# Optimization of the All-D Peptide D3 for Aβ Oligomer Elimination

**DOI:** 10.1371/journal.pone.0153035

**Published:** 2016-04-22

**Authors:** Antonia Nicole Klein, Tamar Ziehm, Markus Tusche, Johan Buitenhuis, Dirk Bartnik, Annett Boeddrich, Thomas Wiglenda, Erich Wanker, Susanne Aileen Funke, Oleksandr Brener, Lothar Gremer, Janine Kutzsche, Dieter Willbold

**Affiliations:** 1 Institute of Complex Systems, Structural Biochemistry (ICS-6), Research Center Jülich, 52425, Jülich, Germany; 2 Institute of Complex Systems, Soft Matter (ICS-3), Research Center Jülich, 52425, Jülich, Germany; 3 Neuroproteomforschung und Molekulare Mechanismen Neurodegenerativer Erkrankungen, Max-Delbrück-Centrum für Molekulare Medizin, Berlin, Germany; 4 Bioanalytik, Fakultät angewandte Naturwissenschaften, Hochschule für angewandte Wissenschaften Coburg, 96450, Coburg, Germany; 5 Institut für Physikalische Biologie, Heinrich-Heine-Universität Düsseldorf, 40225, Düsseldorf, Germany; MedImmune Ltd Research and Development, UNITED KINGDOM

## Abstract

The aggregation of amyloid-β (Aβ) is postulated to be the crucial event in Alzheimer’s disease (AD). In particular, small neurotoxic Aβ oligomers are considered to be responsible for the development and progression of AD. Therefore, elimination of thesis oligomers represents a potential causal therapy of AD. Starting from the well-characterized d-enantiomeric peptide D3, we identified D3 derivatives that bind monomeric Aβ. The underlying hypothesis is that ligands bind monomeric Aβ and stabilize these species within the various equilibria with Aβ assemblies, leading ultimately to the elimination of Aβ oligomers. One of the hereby identified d-peptides, DB3, and a head-to-tail tandem of DB3, DB3DB3, were studied in detail. Both peptides were found to: (i) inhibit the formation of Thioflavin T-positive fibrils; (ii) bind to Aβ monomers with micromolar affinities; (iii) eliminate Aβ oligomers; (iv) reduce Aβ-induced cytotoxicity; and (v) disassemble preformed Aβ aggregates. The beneficial effects of DB3 were improved by DB3DB3, which showed highly enhanced efficacy. Our approach yielded Aβ monomer-stabilizing ligands that can be investigated as a suitable therapeutic strategy against AD.

## Introduction

There are 4.2 million new cases of dementia worldwide each year with Alzheimer’s disease (AD) being the most common cause. Currently, there is no causal treatment for AD available [1, 2].

Intracellular neurofibrillary tangles (NFTs), consisting of hyperphosphorylated tau and extracellular plaques, consisting predominantly of amyloid-β (Aβ), are the major pathological hallmarks of AD. The cleavage of the amyloid precursor protein (APP) by the β- and γ- secretases releases Aβ. Various species of Aβ are formed because the N- and C-terminal cleavage events are non-homogeneous. The most abundant species of Aβ is Aβ(1–40), consisting of 40 amino acids. The second most dominant species is Aβ(1–42) [3, 4].

According to the amyloid cascade hypothesis the aggregation of Aβ is responsible for the development and the progression of AD. Various Aβ aggregate species have been described, including Aβ oligomers and Aβ protofibrils [5]. In particular, soluble, toxic Aβ oligomers are thought to be responsible for damage of synaptic plasticity, formation of free radicals, disequilibrium of intracellular calcium distribution, chronic inflammation and increased phosphorylation of tau [6, 7]. Thus, inhibition of Aβ oligomerization and the elimination of Aβ oligomers are promising treatment strategies for the development of a causal therapy of AD.

We have previously selected the 12mer all-d-enantiomeric peptide D3 via mirror image phage display [8, 9]. *In vitro*, D3 binds to amyloid plaques, reduces Aβ aggregation to regular fibrils, eliminates Aβ oligomers and converts preformed fibrils into non-amyloidogenic, non-fibrillar, non-toxic aggregates [10–14]. *In vivo*, the plaque load and cerebral inflammation of transgenic mice is reduced after injection of D3 into the brain and cognitive impairment of transgenic mice is improved after oral application [10, 15, 16]. The original phage library, from which D3 was selected, coded for about 10^9^ different peptides. A fully randomized 12mer library would contain 20^12^ different peptides. That equals 4 x 10^15^ theoretically possible 12mer sequences and is absolutely impossible to achieve due to limitations in the transformation efficiency during generation of the original phage library. Thus, it can be expected that variation of the D3 sequence will lead to even more potent D3 derivatives.

In the present study, we screened for D3 derivatives with optimized efficiency. To facilitate the screening for various derivatives, peptide microarrays were used because they allow miniaturization, parallelization and automation, and enable high-throughput screenings [17, 18]. In addition, non-natural amino acids and linker groups, like biotin or fluorescein, can be introduced easily.

We screened more than 600 different D3 derivatives for their ability to bind monomeric Aβ and characterized the five most promising candidates by examining their ability to prevent Aβ fibril formation. For further optimization, the most promising D3 derivative DB3 was modified by designing a head-to-tail tandem peptide, called DB3DB3. Both peptides were characterized in more detail regarding their affinity to Aβ monomers and their efficiency to eliminate Aβ oligomers.

## Material and Methods

### Peptides

Aβ(1–42), N-terminally biotinylated Aβ(1–42) and FITC-Aβ(1–42) were purchased from Bachem (Heidelberg, Germany). D3 (RPRTRLHTHRNR), DB1 (RPITRLHTDRNR), DB2 (RPITTLQTHQNR), DB3 (RPITRLRTHQNR), DB4 (RPRTRLRTHQNR) and DB5 (RPITRLQTHEQR) were purchased from JPT (Berlin, Germany). DB3DB3 (RPITRLRTHQNRRPITRLRTHQNR) was purchased from peptides&elephants (Potsdam, Germany). All d-peptides consisted of d-enantiomeric amino acids, were C-terminally amidated and > 95% pure.

### HFIP pretreatment of Aβ(1–42)

For obtaining monomeric Aβ(1–42), N-terminally biotinylated Aβ(1–42) and FITC-Aβ(1–42) were dissolved in 1,1,1,3,3,3-hexafluoroisopropanol (HFIP) overnight to a final concentration of 1 mg/ml and aliquoted. HFIP was evaporated by vacuum concentration (Concentrator 5301, Eppendorf, Germany) for 20 min and the aliquots were stored at -20°C until further usage.

### Peptide Microarrays

#### Pepspot membranes

In a first generation peptide array, every position of the 12 amino acid residue d-peptide D3 was replaced against all 20 naturally occurring amino acids in their d-enantiomeric conformation. The nitrocellulose membrane spotted with these 240 different peptides (JPT, Berlin, Germany) was blocked using TBS pH 7.4 with 10% v/v blocking solution (Roche, Basel, Switzerland) for 2.5 h at room temperature. After 5 min washing with TBS and 0.1% v/v Tween 20 (TBS-T), the membrane was incubated with 5 μM Aβ(1–42) in 10 mM sodium phosphate buffer pH 7.4 for 1 h. The potential of all 240 derivatives to bind monomeric Aβ was measured by applying 6E10 (BioLegend, San Diego, USA, diluted 1:10.000 in TBS pH 7.4) and a horseradish peroxidase (HRP)-conjugated goat anti-mouse antibody (Fisher Scientific, Schwerte, Germany, diluted 1:10.000 in TBS pH 7.4). The membrane was washed with TBS-T pH 7.4 for 2 h. HRP activity was measured after incubation with HRP substrate (Pierce, Waltham, USA) by using a ChemiDoc 200 detection system (Bio-Rad Laboratories, Munich, Germany) and the ImageLab software (Bio-Rad Laboratories, Hercules, Munich, Germany).

#### Pepscan

For the second generation a peptide microarray was produced by Pepscan (Lelystad, Netherlands).

For the Pepscan chip, the peptides were covalently coupled on glass slides in triplicate (spots with diameter of 100 μm). Slides were incubated with 5 μM FITC-Aβ(1–42) in 10 mM sodium phosphate buffer pH 7.4 for 1 h at room temperature with gentle agitation. After incubation, the slides were washed three times with TBS-T for 10 min, three times with water for 10 min and subsequently dried using a stream of nitrogen gas.

Fluorescence intensity of FITC-Aβ(1–42) bound to the peptide spots was measured by a FLA800 fluorescence image system (Fujifilm Medical Systems USA Inc, Stamford, USA) with a slide carrier employing a 473 nm laser for excitation. Digital images were recorded at 5 μm resolution. Fluorescence was analyzed by the software AIDA Array Metrix (Raytest, Staubenhardt, Germany). Signals were integrated for each spot (diameter 80 μm). The background signal was detected from local dot rings with diameter widths of 150 μm and background ring widths of 30 μm, and subtracted from the peptide spot signal.

### Thioflavin T (ThT) Assay

20 μM Aβ(1–42) was mixed with 20 μM Thioflavin T (ThT) and 31 μg/ml DB3 or DB3DB3 in 10 mM sodium phosphate buffer, pH 7.4. The assay was performed using a non-binding 96 well plate (Greiner Bio-One, Frickenhausen, Germany). ThT fluorescence was measured every 15 min at λ_ex_ = 440 nm and λ_em_ = 490 nm in a temperature-controlled plate reader (Polarstar Optima, BMG, Offenburg, Germany) at 37°C with 1 min agitation before every measurement. Each value was background corrected using the ThT fluorescence of a peptide solution without Aβ(1–42).

### Biolayer interferometry (BLI)

BLI experiments were performed using an Octet RED96 instrument (fortéBIO, PALL Life Science, Menlo Park, USA). N-terminally biotinylated Aβ(1–42) was dissolved in HFIP, lyophilized and dissolved in 2 mM aqueous sodium hydroxide (1 mg/ml) in order to destroy any pre-existing aggregates. The Aβ(1–42) solution was neutralized by dilution in running buffer (20 mM sodium phosphate buffer, pH 7.4) to a final concentration of 20 μg/ml and directly immobilized on Super Streptavidin biosensors (SSA) (fortéBIO, PALL Life Science, Menlo Park, USA) to a final depth of 3 nm. Ligand biosensors and reference biosensors were quenched with 20 μg/ml biotin for 7 min.

For K_D_ determinations, the binding of a dilution series of DB3 (200, 100, 50, 25, 12.5, 6.25, 3.125 μM) or DBDB3 (20, 10, 5, 2.5, 1.25, 0.625, 0.3125 μM) was detected in parallel to the ligand biosensors and reference biosensors. A separate buffer cycle was used for double referencing. For evaluation, steady state response levels were plotted against the applied peptide concentrations and fitted according to Langmuir´s 1:1 binding model (Hill function with *n* = 1, OriginPro 8.5G, OriginLab, Northampton, USA).

### QIAD assay

The quantitative determination of interference with Aβ(1–42) aggregate size distribution (QIAD) was performed as described before [14]. In brief, 80 μM Aβ(1–42) was incubated in 10 mM sodium phosphate buffer, pH 7.4 for 4.5 h at 22°C with 600 rpm agitation. Aβ(1–42) aggregation was continued for an additional 40 min with or without DB peptide. The obtained partial size distribution was analyzed by applying density gradient centrifugation. 100 μl of the incubated sample was placed on top of a gradient with 5 to 50% iodixanol (Optiprep, Axis-shield, Oslo, Norway) and separated at 259.000 x g for 3 h at 4°C using an ultracentrifuge (Optima MAX-XP, Beckman Coulter, Brea, USA). Fourteen fractions of 140 μl were taken from top to bottom. The pellet was dissolved by adding 60 μl of 6 M guanidine hydrochloride to the centrifugation tube. After boiling for 5 min, the dissolved pellet sample was collected. The samples were stored at -80°C until further use.

For quantification of the Aβ(1–42) amount in each fraction, reversed-phase high performance liquid chromatography (RP-HPLC) was performed using a Zorbax SB-300 C8 column (Agilent, Böblingen, Germany) connected to an Agilent 1260 Infinity system using 30% (v/v) acetonitrile with 0.1% (v/v) trifluoroacetic acid (TFA) as the mobile phase with a flow of 1 ml/min and a column temperature of 80°C. The applied sample volume was 20 μl. The UV absorption at 214 nm was measured. For quantification of the Aβ(1–42) amount, the area under the peak representing Aβ(1–42) was calculated and the molar concentration was determined using a calibration curve.

For additional control and visualization of the Aβ content in each fraction, a 16% tricine-SDS-PAGE was performed and Aβ(1–42) was visualized by silver staining according to Schagger [19].

### MTT cell viability assay

Rat pheochromocytoma PC12 cells (Leibniz Institute DSMZ, Braunschweig, Germany) were cultivated in DMEM medium supplemented with 10% fetal bovine serum and 5% horse serum. 10,000 cells per well were seeded on collagen-coated 96 well plates (Gibco, Life technology, Carlsberg, USA) and incubated in a 95% humidified atmosphere with 5% CO_2_ at 37°C for 24 h. To yield oligomeric Aβ, monomerized Aβ(1–42) was preincubated for 4.5 h in sodium phosphate buffer at 21°C and 600 rpm agitation. DB peptide was then added at different concentrations and incubated for further 40 min at 21°C and 600 rpm agitation before addition to the PC12 cells. Final concentrations were 1 μM Aβ(1–42) and 0, 2, 1, or 5 μM DB3 or half of the molar peptide concentrations of DB3DB3. The PC12 cells were further incubated for 24 h in 95% humidified atmosphere with 5% CO_2_ at 37°C after adding the Aβ-peptide mixture. Cell viability was then measured using the Cell proliferation Kit I (MTT) (Roche, Basel, Switzerland) according to the manufacturer’s instruction. The absorbance of the formazan product was determined by measuring at 570 nm after subtracting the absorption at 660 nm. For absorption measurements, a Polarstar Optima plate reader (BMG, Offenburg, Germany) was used. All results were normalized to cells that were treated with buffer only.

### Aβ Aggregation inhibition ELISA

Freshly dissolved monomeric Aβ(1–42) (400 nM in 500 mM Tris-buffer pH 7.4) was incubated with and without DB peptides in different concentrations (0.01, 0.05, 0.1, 0.5, 1, 5, 10, 50 and 100 μM for DB3 or half of the molar concentrations in case of DB3DB3) in a humidity chamber for 23 h at 37°C. The aggregation was analyzed by an enzyme-linked immunosorbent assay (ELISA). NP27 antibody in bicarbonate/carbonate buffer was used to coat the ELISA plate overnight. Then the plate was washed in PBS-Tween buffer (1x PBS + 0.05% Tween) and blocked for 2 h at room temperature with 5% casein buffer. After washing, Aβ aggregate solutions were added to the plate and incubated for 1 h at room temperature. The plate was washed again and bound Aβ aggregates were detected by biotinylated 6E10/HRP-avidin mediated immunoreaction (BioLegend, San Diego, CA USA) using TMB as detection reagent. Each value was background corrected which were derived from ELISA of samples without capture antibody and normalized to the control without peptide (0% no inhibition, 100% full inhibition). Mean value and standard error were calculated from three independent experiments. EC_50_ was calculated by fitting the data to a logistic dose response function.

### Aβ Aggregate disassembly ELISA

Freshly dissolved monomeric Aβ(1–42) (400 nM in 500 mM Tris-buffer pH 7.4) was incubated in a humidity chamber for 22 h at 37°C in order to preform Aβ(1–42) aggregates. These preformed aggregates were coincubated with d-peptide in different concentrations (0.01, 0.05, 0.1, 0.5, 1, 5, 10, 50, 100 μM for DB3 or with half of the molar concentrations in case of DB3DB3) for additional 22 h at 37°C. The content of Aβ aggregates was measured and evaluated in the same way as the aggregation inhibition ELISA.

### Transmission electron microscopy (TEM)

10 μM of freshly dissolved monomeric Aβ(1–42) was incubated in 10 mM sodium phosphate buffer pH 7.4 with or without DB peptide in equal molar ratios for 24 h at 37°C. Afterwards, 20 μl of the samples were absorbed on formval/carbon coated copper grids (S162, Plano, Wetzlar, Germany) for 3 min, washed three times with water and negative stained with 1% v/v uranylacetate for 1 min. The images were acquired using a Libra 120 electron microscope (Zeiss, Oberkochen, Germany) at 120 kV.

### Statistical analysis

Statistical analysis was performed using the Origin 8.5 (OriginLab Cooperation, Northampton, USA) software package.

## Results

### Screening for optimized D3 derivatives using peptide microarrays

**Fig 1:**
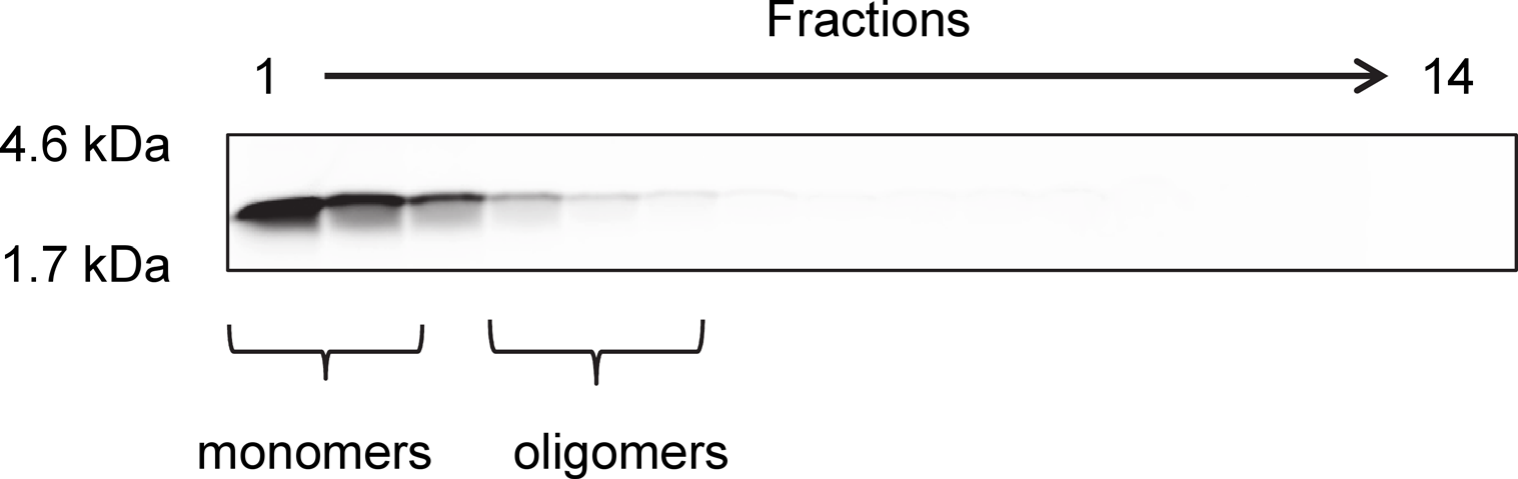
Aggregation state of monomeric Aβ(1–42) after 1 h incubation. For optimization of D3 with peptide microarrays, the peptide microarrays were incubated with 5 μM initially monomeric Aβ(1–42) for 1 h at room temperature. The aggregation state of this Aβ preparation was analyzed by density gradient centrifugation followed by 16% Tricine-SDS-PAGE. FITC-Aβ(1–42) was detected via FITC fluorescence and was only detectable in the first four lanes, which represent mainly monomeric and oligomeric FITC-Aβ [14].

**Fig 2:**
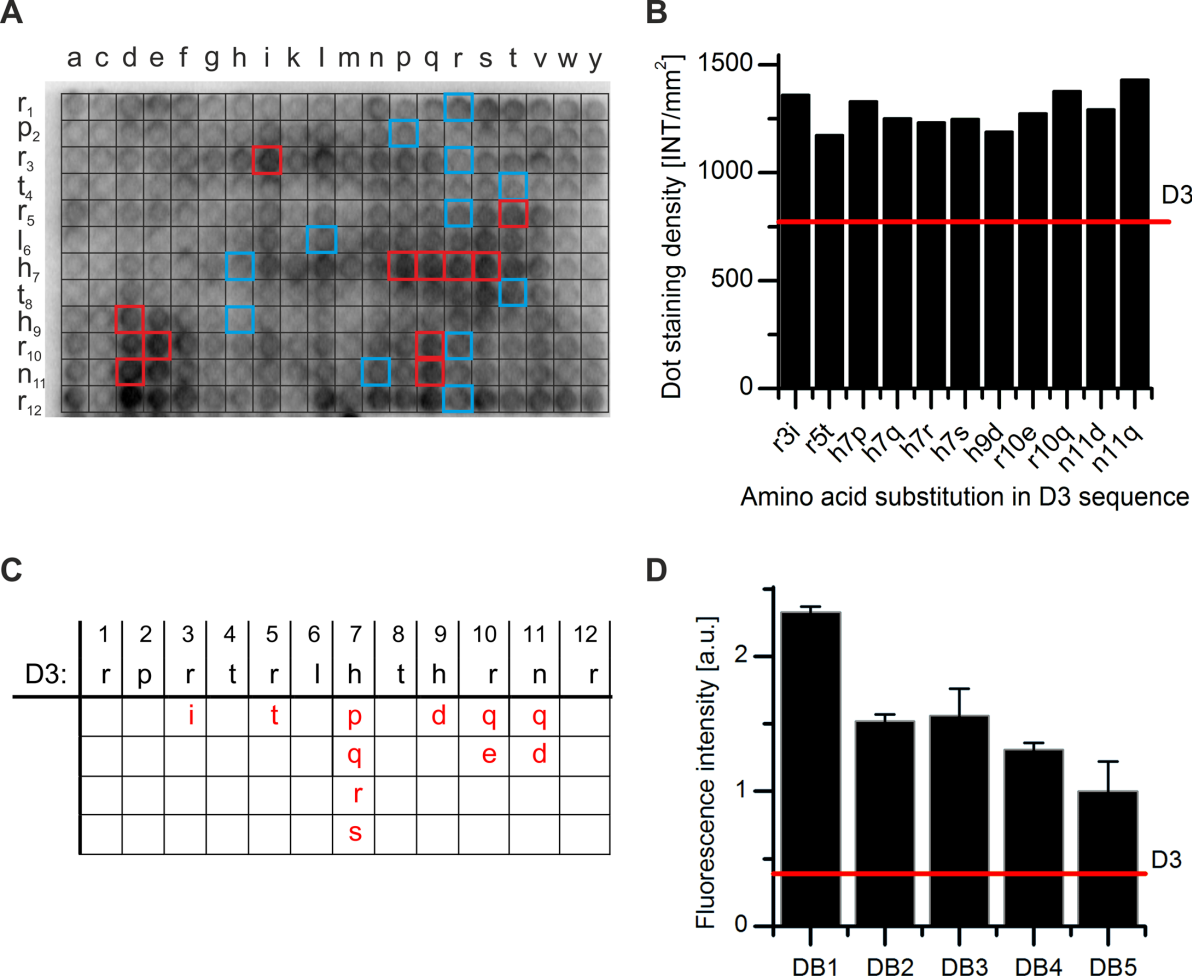
Selection of DB1 to DB5 based on two cycles of peptide microarray based screenings. A) Promising replacements in the sequence of D3 were selected via PepSpots peptide array. Binding of monomeric Aβ(1–42) to spotted D3 derivatives was detected using the Aβ antibody 6E10 and a HRP-labeled secondary antibody. Several of the dots with the highest staining density, representing the most promising single replacements, are marked in red. The original D3 controls are indicated in blue. B) The HRP-intensity was evaluated by the staining density of the peptide dots and plotted against the amino acid substitutions. Eleven promising substitutions that showed > 1.5 times increase in binding to monomeric Aβ(1–42) when compared with that of D3, were chosen for a second generation peptide microarray. The red line represents the mean dot staining intensity of D3. C) Schematic overview of the first generation microarray output. D) Binding of FITC-Aβ(1–42) to the peptides DB1 to DB5. The binding of FITC-Aβ(1–42) to the spotted peptides was analyzed by measuring the FITC-fluorescence intensity. All intensities were background corrected. The signal intensities of the top five peptides were plotted. The red line represents the mean fluorescence intensity of D3.

We identified previously the Aβ oligomer eliminating d-enantiomeric peptide D3 via mirror image phage display [10, 16]. A possible explanation of the efficiency of D3 is that it binds to and stabilizes Aβ monomers within the various equilibria with Aβ oligomers and other Aβ assemblies. In order to identify more efficient derivatives, a systematic optimization of D3 regarding its binding affinity to monomeric Aβ(1–42) was performed using a two-step procedure. For the first step, every position of the original peptide D3 was replaced against each of the 19 other proteinogenic amino acids residues in their D-enantiomeric form. These 20x12 different peptides were spotted on a Pepspot membrane (JPT, Berlin, Germany) and the binding of monomeric Aβ(1–42) was measured according to the procedure described in the material and method part. To verify that the applied Aβ(1–42) was mainly monomeric even after the 1 h incubation period, a density gradient ultracentrifugation run was done with an aliquot of the applied Aβ(1–42) that was incubated 1 h, too. Even after 1 h incubation, only a very small fraction of Aβ(1–42) was found in fractions 4 and higher (Fig 1) indicating that by far the largest part of the Aβ is still monomeric and thus can be found in fractions 1 to 3 [14]. After washing, the amount of bound Aβ was determined by the Aβ-specific antibody 6E10, which is known to bind all Aβ species regardless of their assembly state, and a secondary detection antibody (Fig 2A). The amino acid substitutions that yielded the highest Aβ binding activity, as measured by the dot staining density, were chosen for further combinations in the second round (Fig 2B and 2C). In particular, the substitutions R3I, R5T, H9D, R10Q, R10E, N11Q and N11D were found to bind more favorably to Aβ monomers. The residue H7 showed the highest potential for further improvement, because most substitutions at this position yielded higher affinity towards Aβ monomers. H7P, H7Q, H7R and H7S were selected as the most promising substitutions for H7. Substitution of R12 was excluded from the analysis because a minimum number of arginines has been recognized to be responsible for the superior pharmacokinetic properties of D3 [20]. Interestingly, nine of the eleven substitutions were located in the C-terminal half of D3 at positions 7, 9, 10 and 11.

For the second round of optimization, every possible combination of the eleven single residue replacements R3I, R5T, H7P, H7Q, H7R, H7S, H9D, R10Q, H10E, N11Q and N11D, were combined to yield 360 different peptides, which were spotted on a glass chip (Pepscan, Lelystad, Netherlands) (Fig 2C). To compare their binding activities to monomeric Aβ, the peptide microarrays were incubated with freshly dissolved monomeric FITC-Aβ(1–42) and fluorescence intensities of the Aβ-peptide interactions were measured. Five peptides that showed tight binding to Aβ monomers as deduced from high FITC fluorescence intensities were chosen for further *in vitro* characterization (Fig 2D and Table 1). The fluorescence intensities of these D3 derivatives, termed DB1 to DB5, were up to six times higher when compared with the fluorescence intensity obtained with D3 (Fig 2D). As shown in Table 1, the sequences of DB1 to DB5 had two to four amino acid substitutions to the original D3 template.

**Table 1:** Amino acid sequences of D3 and DB1 to DB5.

name	sequence
D3	RPRTRLHTHRNR
DB1	RP**I**TRLHT**D**RNR
DB2	RP**I**T**T**L**Q**TH**Q**NR
DB3	RP**I**TRL**R**TH**Q**NR
DB4	RPRTRL**R**TH**Q**NR
DB5	RP**I**TRL**Q**TH**EQ**R

All amino acids of the peptides are d-enantiomeric and their C-termini are amidated. The amino acid substitutions made to the D3 template are indicated in bold.

### The influence of DB3 and DB3DB3 on Aβ fibril formation

To investigate the influence of DB1 to DB5 on Aβ fibril formation, a Thioflavin T (ThT) assay was performed. In an aqueous environment the benzothiazole dye has a low fluorescence. Upon interaction with regularly formed amyloid fibrils the fluorescence signal is significantly enhanced and excitation and emission maxima shift from 385 and 445 nm to 450 and 490 nm, respectively. The emission at 490 nm is directly proportional to the quantity of amyloid fibrils. Fibril formation of Aβ can be followed in real time by measuring the ThT fluorescence [21–23].

**Fig 3:**
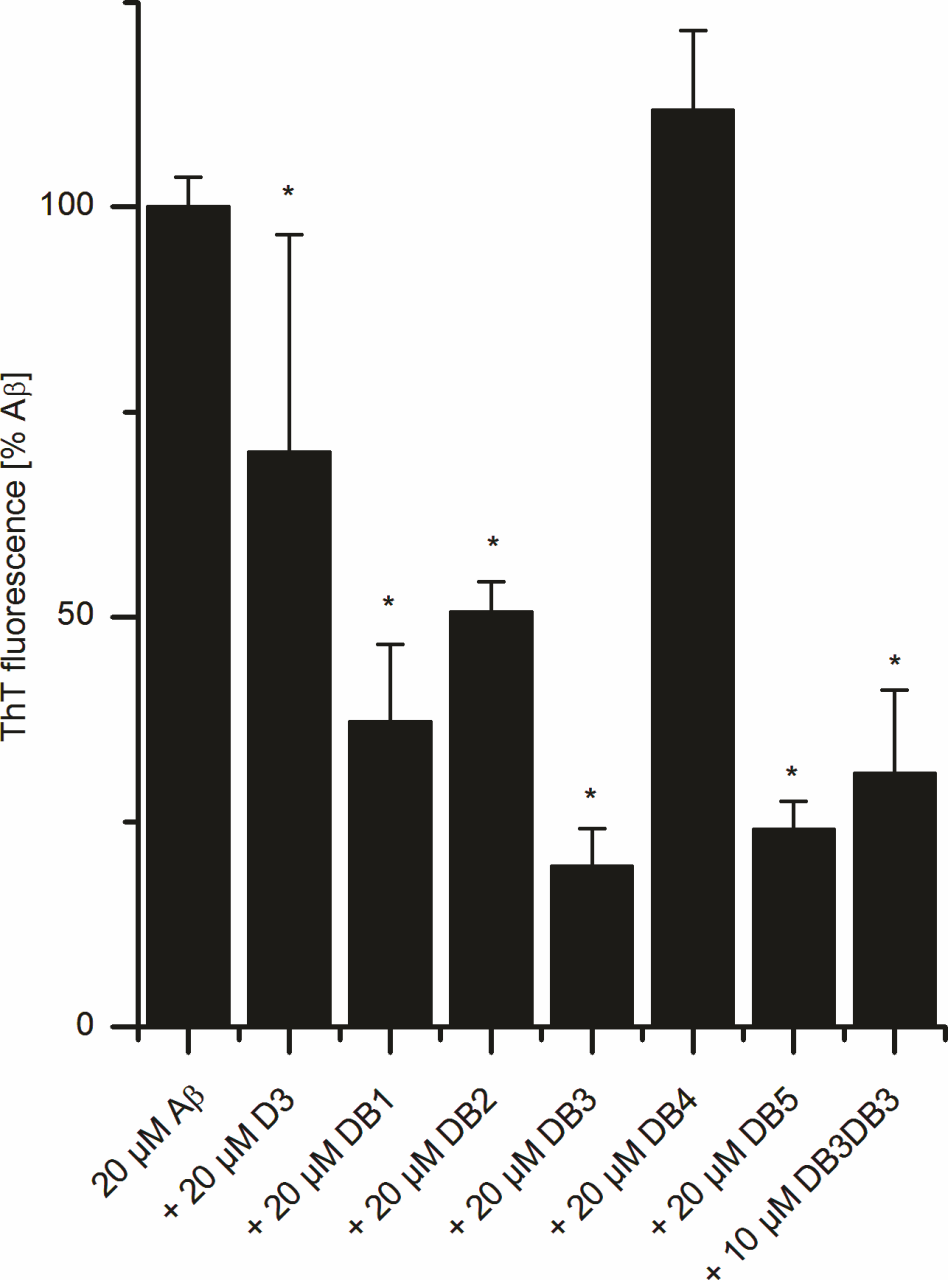
Thioflavin T fibril formation assay. 20 μM Aβ(1–42) was mixed with 20 μM DB1 to DB5 or 10 μM DB3DB3 and the ThT fluorescence was monitored. Aβ(1–42) without peptide addition was taken as the control. The ThT fluorescence of all samples were compared after 5 h, where the control, Aβ(1–42) only, reached its maximum in fluorescence emission. The Mann-Whitney-U-test was used for statistical analysis. * *p* < 0.05; ** *p* < 0.01; *** *p* < 0.001.

Therefore, the inhibitory effects of the d-peptides DB1 to DB5 and D3 were investigated by co-incubating these peptides with Aβ(1–42) and performing the ThT assay. Fluorescence emission data were compared after 5 h incubation, because after this period the Aβ(1–42) control (without added peptide) reached its fluorescence maximum. As shown in Fig 3, D3 inhibited Aβ(1–42) fibril formation by 30%, whereas DB3 inhibited the Aβ(1–42) fibril formation by 80%, DB5 by 76%, DB1 by 63% and DB2 by 49% when compared to the control (Fig 3). Surprisingly, DB4 had no effect on the fibril formation of Aβ.

The results of the ThT assay indicate that DB3 is the most promising peptide according to the inhibitory effect of fibril formation. Therefore, we selected DB3 for further in vitro studies. As a further potential optimization step of the DB peptides, we wanted to investigate the impact of avidity. Although the DB peptides were selected for monomer binding, elimination of toxic oligomer species might require contacting monomer units within these oligomers. In order to make use of the multivalence of oligomers, multivalent DB3 could possibly have increased efficiency in oligomer elimination. As the simplest multivalent DB3 peptide, we designed a head-to-tail tandem peptide of DB3, named DB3DB3. In contrast to other divalent DB3 molecules, e.g. head-to-head or tail-to-tail orientations, the head-to-tail tandem of DB3 contains only peptide bonds between the amino acid residues and is thus easily accessible by standard peptide synthesis. As shown in Fig 3, 10 μM DB3DB3 inhibited the formation of ThT-positive aggregates as efficiently as 20 μM DB3.

### Binding affinities of DB3 and DB3DB3 to Aβ(1–42) monomers

**Fig 4:**
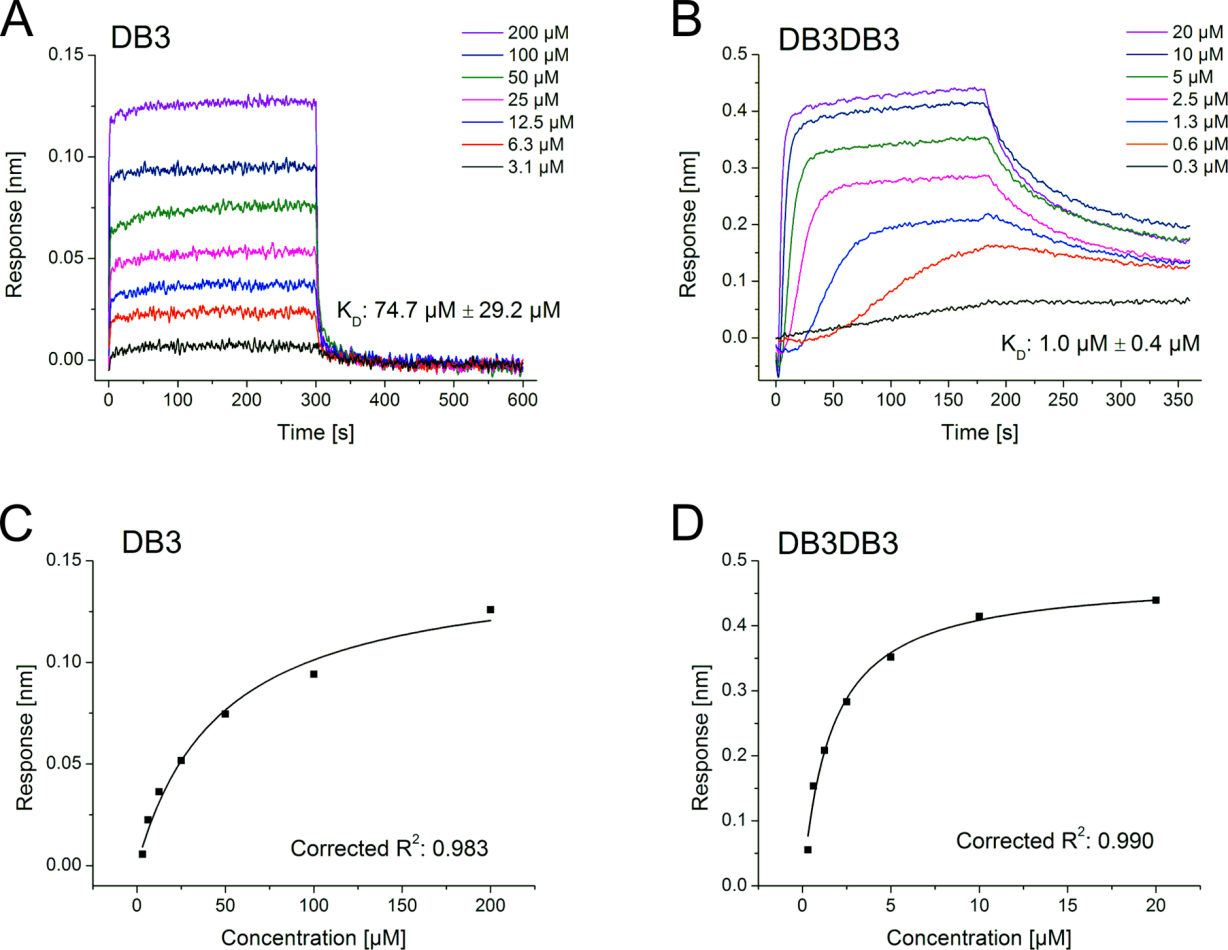
K_D_ determination of DB3 and DB3DB3 to monomeric Aβ using biolayer interferometry (BLI). N-terminally biotinylated Aβ(1–42) monomers were immobilized on streptavidin biosensors and the binding of DB3 and DB3DB3 was detected. Representative double referenced sensorgrams of a dilution series of DB3 (A) and DB3DB3 (B) are shown, including the equilibrium dissociation constants (K_D_) as means ± SD of data recorded in triplicate. For steady state analysis Langmuir´s 1:1 binding model was applied. Representative fits of DB3 (C) and DB3DB3 (D) are depicted with the corresponding corrected *R* ^2^.

For further characterization of DB3 and DB3DB3, the equilibrium dissociation constants (*K*_D_) of the d-peptides were determined for their interaction with Aβ(1–42) monomers using biolayer interferometry (BLI) (Fig 4). For DB3, a *K*_D_ value of 75 μM was determined, whereas for the designed dimer peptide DB3DB3 a *K*_D_ value of 1 μM was obtained. Therefore, the binding affinity to Aβ(1–42) monomers was enhanced by 75-fold for the dimeric version of DB3.

### Aβ aggregation inhibition by DB3 and DB3DB3

**Fig 5:**
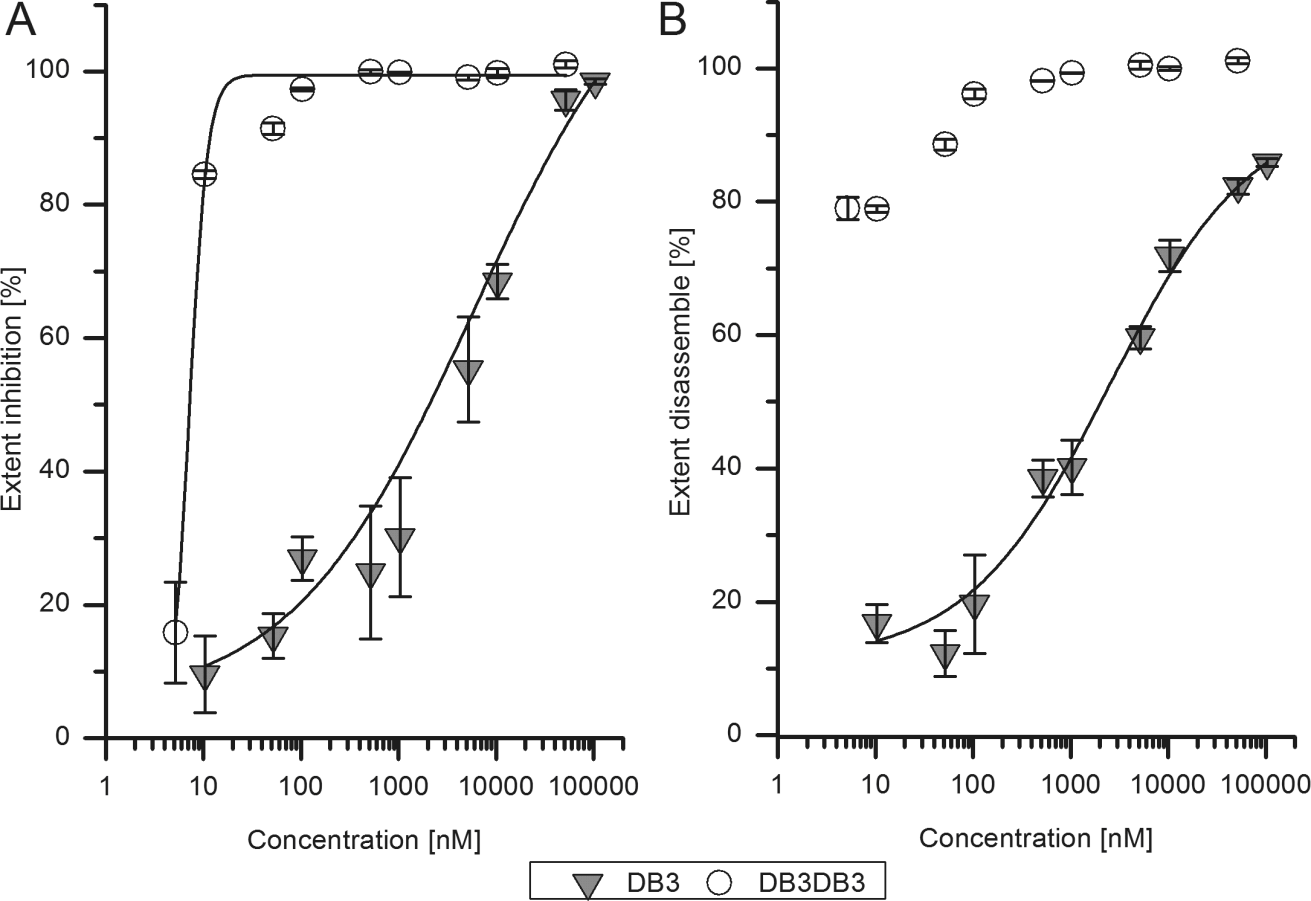
Inhibition of Aβ fibril formation and Aβ aggregation disassemble by DB3 and DB3DB3. A) Monomeric Aβ(1–42) (400 nM) was mixed with different concentrations of DB3 (0.01, 0.05, 0.1, 0.5, 1, 5, 10, 50, 100 μM) and the aggregation state of Aβ was analyzed using an Aβ aggregate specific ELISA. For DB3DB3 half of the molar concentrations compared to DB3 were used. Aβ without DB3 and DB3DB3 addition was taken as control. For DB3 an EC_50_ of 6 μM was calculated using a logistic fit model. DB3DB3 inhibited the formation of Aβ fibrils more efficiently with an EC_50_ of 7 nM. B) The disassembly properties of DB3 and DB3DB3 were measured using an Aβ aggregation specific ELISA. Monomeric Aβ(1–42) (400 nM) was preincubated in order to form fibrils and mixed with nine different concentrations of DB3 (0.01, 0.05, 0.1, 0.5, 1, 5, 10, 50, 100 μM). For DB3DB3, the molar concentrations were half those used for DB3. For DB3 an EC_50_ of 2.5 μM was determined. DB3DB3 disassembled Aβ aggregates at the lowest concentration (10 nM). Thus, the EC_50_ could not be determined, but is < 10 nM. All data were determined in triplicate. The Mann-Whitney-U-test was performed for statistical analysis. * *p*< 0.05; ** *p* < 0.01; *** *p* < 0.001

To confirm and further investigate the efficiency of DB3 and DB3DB3 activity on Aβ aggregation, an aggregation inhibition ELISA was performed. Initially monomeric Aβ(1–42) was incubated with different concentrations of DB3 or DB3DB3 and the Aβ(1–42) aggregates were specifically detected via ELISA. DB3 inhibits Aβ aggregate formation with an EC_50_ of 6 μM, whereas DB3DB3 inhibits Aβ aggregate formation with an EC_50_ of 8 nM (Fig 5A). Thus, DB3DB3 is 1000-fold more efficient in inhibiting Aβ aggregation when compared with that of DB3. Furthermore, the aggregation inhibition ELISA showed that the fibrillization of Aβ(1–42) was almost inhibited completely at a peptide concentration of 20 μM DB3 and 10 μM DB3DB3.

### Aβ aggregates disassembly ability of DB3 and DB3DB3

The Aβ aggregate disassembly ELISA was used to analyze the effect of DB3 and DB3DB3 on preformed Aβ aggregates (Fig 5B). Preformed Aβ(1–42) aggregates were co-incubated with different concentrations of DB3 or DB3DB3 for 24 h. The Aβ aggregates specific ELISA was used to quantify Aβ aggregates. The raw data were normalized to Aβ aggregates without peptide. The results of measured Aβ aggregates normalized to the Aβ control were plotted against the peptide concentration. The EC_50_ was calculated by a logarithmic dose response function.

The EC_50_ value using D3 to dissemble the Aβ aggregates was 2.5 μM; however, the value for DB3DB3 could not be determined because the Aβ aggregates were already disassembled at very low peptide concentrations, i.e., addition of 10 nM DB3DB3 to 400 nM Aβ gave 80% disassembly of the Aβ aggregates when compared to Aβ in the absence of peptide.

### Elimination of Aβ oligomers

**Fig 6:**
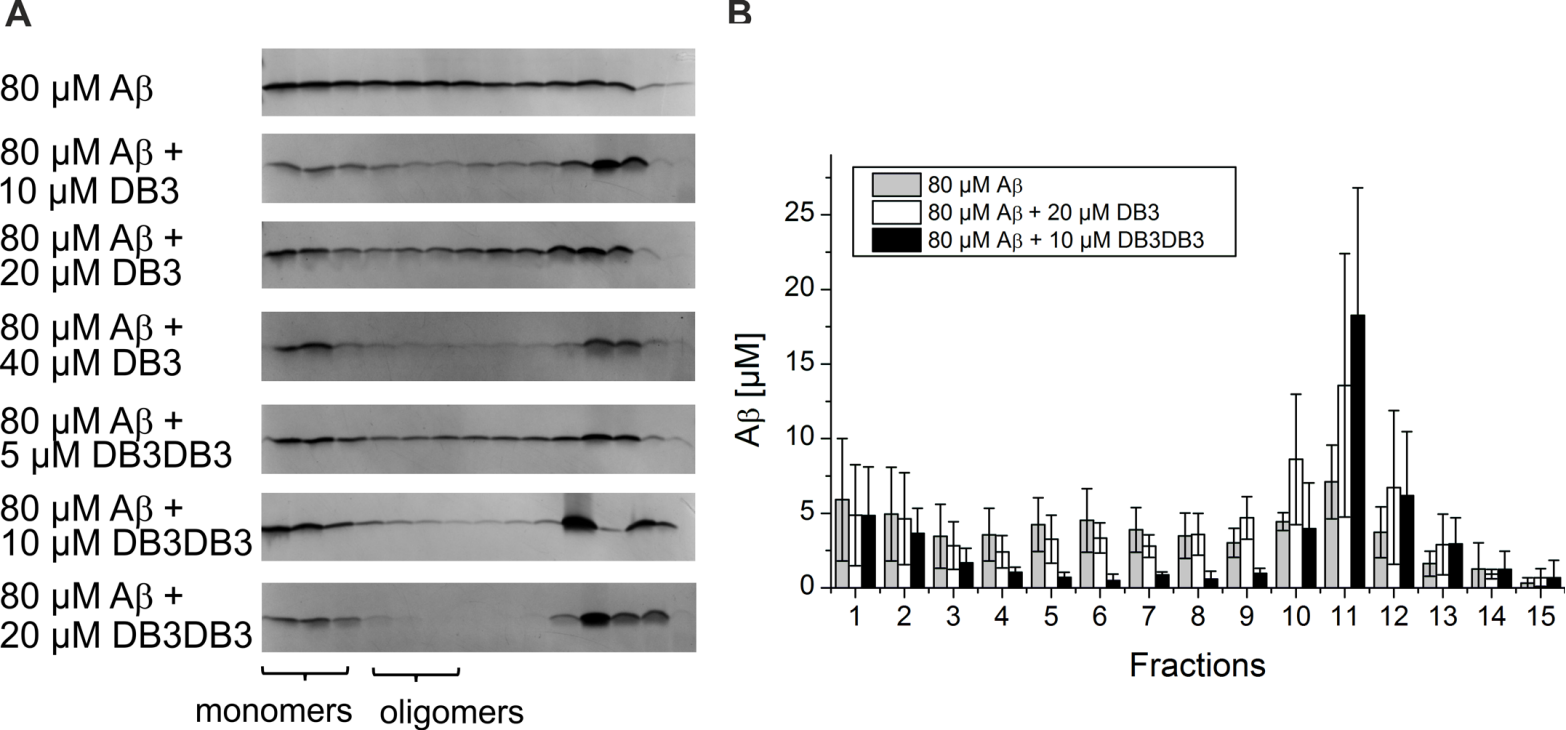
Effect of DB3 and DB3DB3 on different Aβ aggregation species. A) Analysis of Aβ(1–42) aggregation species with density gradient centrifugation and followed by analysis using silver-stained Tricine-SDS-PAGE to analyze the influence of DB3 and DB3DB3 on the distribution of Aβ assemblies. B) Quantification of Aβ(1–42) by RP-HPLC. All data were recorded in triplicate.

Aβ oligomers are the main toxic species and are discussed to be responsible for development and progression of AD [7]. A promising therapeutic approach is the elimination of Aβ oligomers. We have previously established an assay that determines quantitatively the Aβ oligomer elimination efficiency of a given substance (QIAD assay) [14]. By applying this assay the Aβ(1–42) oligomer elimination efficacy of DB3 and DB3DB3 (Fig 6) was determined. Incubation of 80 μM monomeric Aβ(1–42) for 4.5 h led to the formation of a mixture of Aβ monomers (fractions 1–2), oligomers (fractions 4–6) and larger aggregates (other fractions) (Fig 6A). Addition of DB3 at different concentrations resulted in the elimination of Aβ oligomers in a concentration-dependent manner. Addition of 40 μM DB3 to Aβ oligomers yielded near complete elimination of the oligomers. In comparison, addition of 20 μM DB3DB3 to 80 μM Aβ eliminated the oligomer species completely. Moreover, the content of Aβ oligomers was also strongly reduced when applying 10 μM DB3DB3.

RP-HPLC was used to quantify the Aβ oligomer elimination efficiency (Fig 6B). 20 μM DB3 reduced the amount of Aβ oligomers by ~27% compared with that of the Aβ only control. 10 μM DB3DB3 reduced the content of Aβ oligomers by 82%, which is a significant improvement over the DB3 result. The content of large co-precipitates increased and represented the content of Aβ oligomers that were eliminated. The content of monomeric Aβ was not affected by DB3 and DB3DB3. Thus, DB3 and DB3DB3 eliminated Aβ oligomers without affecting the monomers and shifted the equilibrium from oligomeric Aβ to larger Aβ aggregates.

### Reduction of Aβ toxicity

**Fig 7:**
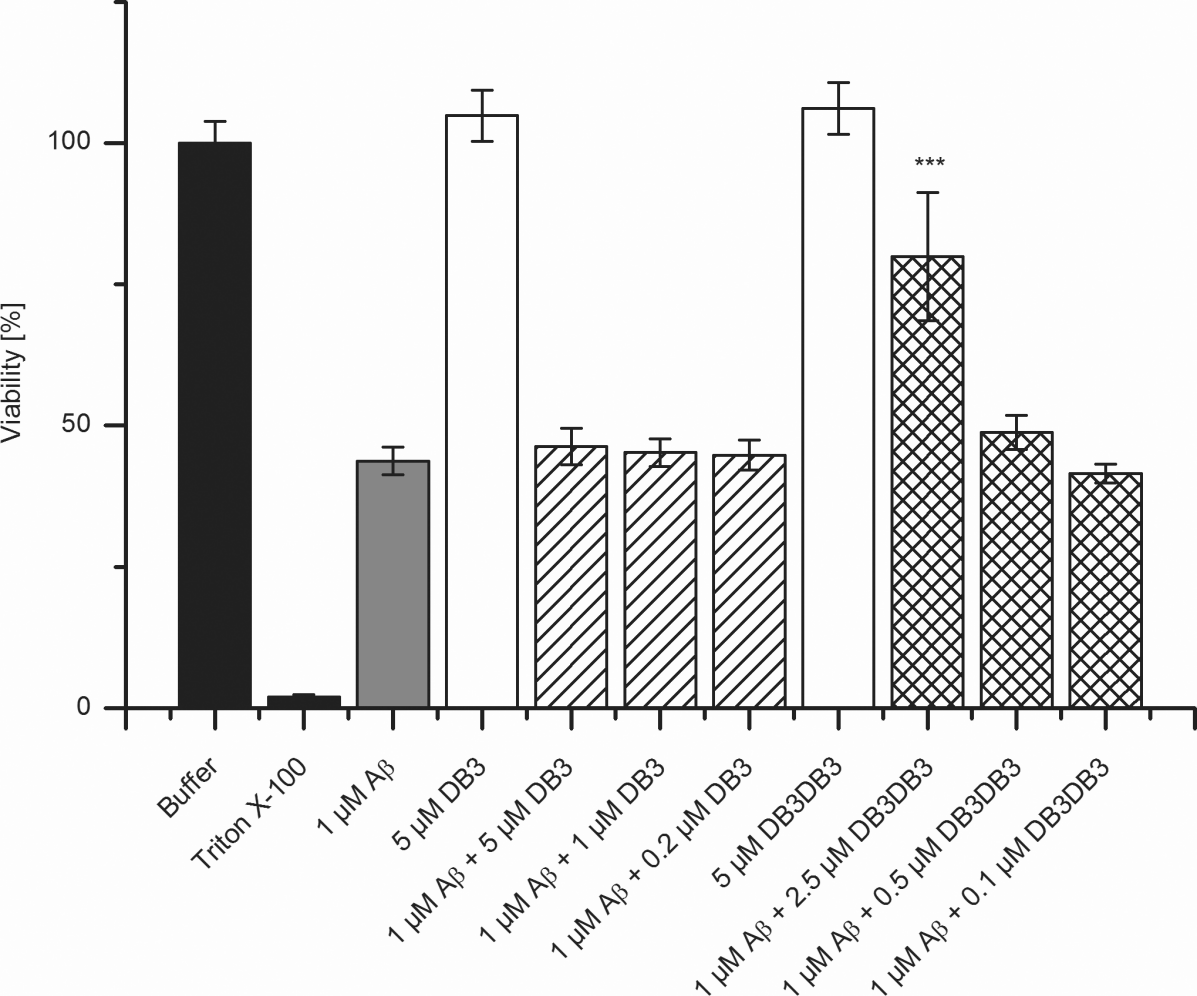
Influence of DB3 and DB3DB3 on Aβ-introduced cytotoxicity. The cell viability assay was performed using PC12 cells in a MTT test. Therefore, Aβ(1–42) was preincubated for 4.5 h and further coincubated with DB3 or DB3DB3 for 40 min. The cells were incubated for 24 h with the Aβ(1–42)-peptide mixture or Aβ(1–42) alone as a control. The absorption of buffer treated cells was set to 100% cell viability. The cell viability of cells treated with Aβ and DB3 or DB3DB3 were compared with cells treated with Aβ only. The Mann-Whitney-U-test was used for statistical analysis. * ***p*** < 0.05; ** ***p*** < 0.01; *** ***p*** < 0.001.

The MTT assay with rat PC12 cells was performed to analyze the influence of DB3 and DB3DB3 to Aβ-induced cytotoxicity (Fig 7). Monomeric Aβ(1–42) was pre-incubated for 4.5 h to yield Aβ oligomers. After additional co-incubation with DB3 or DB3DB3 for 40 min, the mixture was added to PC12 cells and cell viability was analyzed after 24 h using the MTT assay.

In the absence of peptides a solution of 1 μM Aβ reduced the PC12 cell viability to 44% (Fig 7). In contrast, neither DB3 (5 μM) nor DB3DB3 (5 μM) exhibited any effect on PC12 cell viability, which indicates that both peptides are not toxic at the applied concentration. Addition of DB3 to 1 μM pre-incubated Aβ over the concentration range of 0.2 to 5 μM did not significantly increase cell viability. However, a significant concentration-dependent increase of cell viability was observed in the presence of 0.1 to 2.5 μM DB3DB3 in a concentration dependent manner up to 80% (Fig 7). Thus, DB3DB3 was able to inhibit Aβ-induced cytotoxicity.

### Morphology of co-incubated Aβ

To analyze the morphology of Aβ co-complexes with DB3 and DB3DB3, initially monomeric Aβ(1–42) was incubated with DB3 and DB3DB3 for 24 h and TEM analysis performed. For TEM analysis, the samples were absorbed onto formval/copper grids and negatively stained using uranyl acetate.

**Fig 8:**
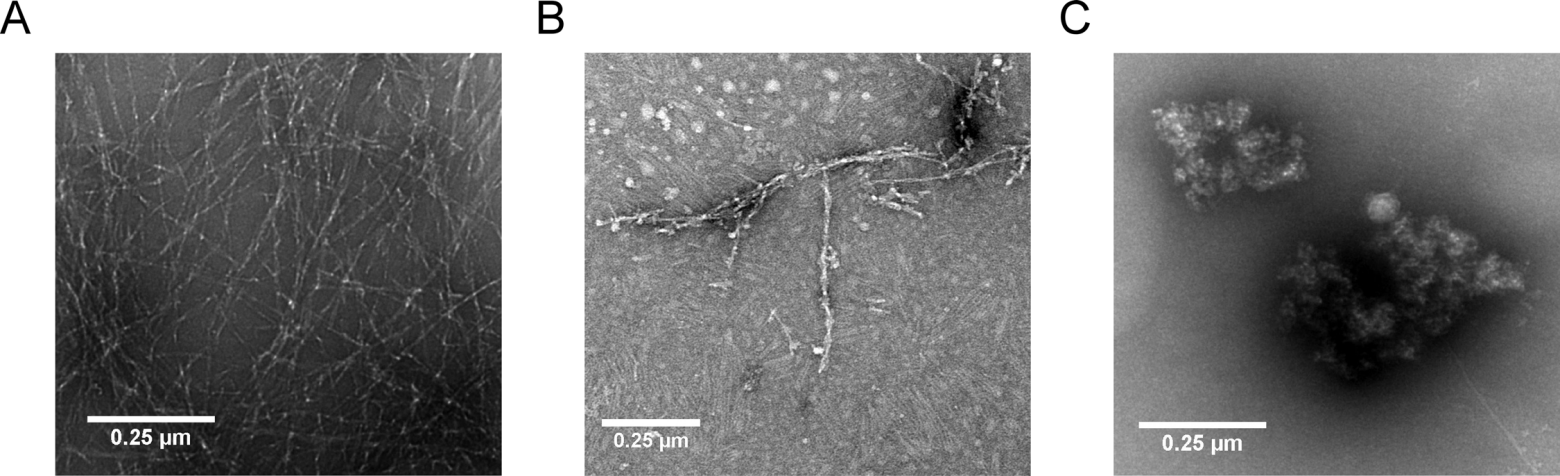
TEM of Aβ-DB3 and -DB3DB3 co-complexes. 10 μM initial monomeric Aβ(1–42) without (A) and with 10 μM DB3 (B) or 5 μM DB3DB3 (C) were coincubated for 24 h. Subsequently, the samples were absorbed onto formval/carbon coated copper grids and negative stained with 1% uranyl acetate. The images were obtained using a transmission electron microscope (TEM). Scale bar: 0.25 μm.

Aβ formed large meshes of fibrils after 24 h incubation (Fig 8A). Co-incubation of Aβ with DB3 in an equal molar ratio resulted in the formation of substantially fewer and shorter fibrils (Fig 8B), which is in perfect accordance with the observation from the ThT assay that DB3 was able to reduce fibril formation by 80%. Co-incubation of Aβ with DB3DB3 yielded huge amorphous co-precipitates, which did not contain any fibrillar structures (Fig 8C). Obviously, at least under these artificially high concentrations, DB3DB3 did not yield mostly Aβ monomers, but high-molecular-weight non-fibrillar co-precipitates with Aβ (Fig 8C), as also observed in the QIAD assay (Fig 6).

## Discussion

Currently, there is no causal therapy for Alzheimer’s disease (AD). *In vitro* and *in vivo* studies showed that Aβ oligomers play an important role in the progression of AD [7]. Therefore, elimination of these toxic Aβ oligomers is a promising strategy to retard AD. Using peptide microarrays, we have optimized the amino acid sequence of the well-characterized d-enantiomeric Aβ oligomer-eliminating peptide D3. The most promising D3 derivatives, DB1–DB5, exhibit two to four different amino acids compared with D3. Besides DB4, the DB peptides have a lower net charge than D3, due to the substitution of R3, R5 and R10, and the introduction of negatively charged amino acids. The substitution of R10Q within DB4 was charge compensated through the substitution H7R. Additional substitutions that had no effect to the net charge were H7Q and N11Q. Combination of single amino acid residue replacements within D3 that each showed slightly enhanced binding capabilities to monomeric FITC-Aβ (Fig 2B) ultimately yielded D3 derivatives that are characterized by significantly enhanced binding capabilities to monomeric FITC-Aβ (Fig 2D). Increased binding capabilities to monomeric FITC-Aβ were solely deduced from fluorescence intensities. Although we cannot prove that this assumption holds true for each peptide spot, it allowed us to pre-select the five most promising D3 derivatives that subsequently were compared to each other.

The inhibition efficacy of Aβ aggregation by DB1 to DB5 was investigated. DB3 was found to be the most promising D3 derivative that inhibited the Aβ aggregation by up to 80%. In contrast, D3 inhibited Aβ aggregation by only 30%. Considering the results of the ThT assay, DB3 was chosen for further *in vitro* characterization. BLI analysis revealed that DB3 interacts with Aβ monomers with a binding affinity of 75 μM, and an ELISA showed that DB3 inhibits the formation of Aβ aggregates with an EC_50_ of 6 μM.

The d-enantiomeric peptides D3 and DB1–DB5 were developed for elimination of Aβ oligomers. This was tested by the QIAD assay [14]. The addition of DB3 to Aβ reduced Aβ oligomers by 28%. Aβ monomers, which are assumed to have neuroprotective functions [24], were not affected. The reduction of oligomeric Aβ resulted in an increase of large amorphous Aβ co-precipitates. TEM images showed that these aggregates possess a higher density. Typical Aβ fibrils, which are linear, unbranched and 5 to 10 nm wide [25], were not visible. Additionally, DB3 was able to disassemble preformed Aβ aggregates.

Since Aβ oligomers are a multivalent target, the divalent tandem peptide DB3DB3 was expected to be significantly more effective. DB3DB3 showed a 75-fold higher affinity for Aβ monomers. This increase in affinity resulted in an increase in the inhibition of Aβ fibrilation and an increased reduction in Aβ-induced cytotoxicity. Additionally, in the Aβ aggregation inhibition ELISA, DB3DB3 yielded an EC_50_ that was 1000-fold lower when compared with that of DB3. DB3DB3 was also able to efficiently eliminate Aβ oligomers as shown in the QIAD assay. Interestingly, DB3 and DB3DB3 did not significantly affect the Aβ monomer content. TEM images showed that these aggregates were not fibrillary structured.

In summary, our *in vitro* data show that the D3 derivative DB3 and its tandem version DB3DB3 were highly efficient at reducing Aβ oligomer content in samples. In particular, the tandem peptide DB3DB3 yielded a significant optimization step when compared with the original peptide DB3. *In vivo* studies will show whether the new compounds’ *in vitro* properties can be translated into enhanced therapeutic activity in AD animal models.
