# Correction: Optimization of the All-D Peptide D3 for Aβ Oligomer Elimination

**DOI:** 10.1371/journal.pone.0158960

**Published:** 2016-07-06

**Authors:** The PLOS ONE Staff

## Notice of Republication

This article was republished on June 21, 2016, to correct low resolution figures in the PDF of the article that were introduced during composition. The publisher apologizes for the errors. The republication also corrects an error in the image for Fig 4 in the HTML and PDF versions of the article.
